# Effect of land-use change along altitudinal gradients on soil micronutrients in the mountain ecosystem of Indian (Eastern) Himalaya

**DOI:** 10.1038/s41598-021-93788-3

**Published:** 2021-07-12

**Authors:** Burhan U. Choudhury, Meraj A. Ansari, Mahasweta Chakraborty, Thounaojam T. Meetei

**Affiliations:** 1 Division of System Research and Engineering, ICAR Research Complex for NEH Region, Umiam, Meghalaya 793 103 India; 2 ICAR Research Complex for NEH Region, Manipur Centre, Imphal, Manipur 795 004 India

**Keywords:** Environmental sciences, Solid Earth sciences

## Abstract

Management of soil micronutrients for better crop production needs a sound understanding of their status and causes of variability. This is more relevant for acid soils of the mountain ecosystem of Eastern Himalaya (Northeast India). We assessed the status, and the effect of land uses along altitudinal gradients (14 to 4090 masl) on soil properties and micronutrient concentrations (DTPA extractable Fe, Mn, Cu, and Zn) across the region. Soils varied widely in micronutrient concentrations: Fe from 0.665 to 257.1 mg kg^−1^ while Mn, Cu, and Zn from traces to 93.4, 17.1, and 34.2 mg kg^−1^, respectively. On conversion of evergreen forests (EF) to upland agriculture (Shifting—SC and Settled—SA) and plantation (PH), Mn, Cu, and Zn concentrations decreased significantly from 30.5, 1.74, and 2.13 mg kg^−1^ to 6.44–17.8, 0.68–0.81, and 1.06–1.42 mg kg^−1^, respectively. Grassland (GL) and lowland paddy (LP) had comparable Fe, Mn, and Cu concentrations (except Zn). Degradation of EF to scrubland (SL) recorded the lowest Mn (5.91 mg kg^−1^), Cu (0.59 mg kg^−1^), and Zn (0.68 mg kg^−1^) concentrations. Fe concentration was however increased in degraded SL (+ 73%) over EF (48.7 mg kg^−1^). The distribution of micronutrients among the land uses was inconsistent and followed the order: (i) Fe: SL > PH > LP > EF > GL > SC > SA, (ii) Mn: EF > GL > LP > PH > SC > SA > SL; (iii) Cu: EF > GL > LP > SC > SA = PH > SL; and (iv) Zn: GL > EF > LP > SC > SA > PH > SL. Four micronutrients responded differently and followed a non-linear, 6th—order polynomial trend along the altitudinal gradients (< 500 to 4100 masl). Peak concentrations of Fe, Mn, and Cu were recorded at 1001–2000 m while Zn was recorded at > 4000 masl. The variability (54–64%) in soil micronutrients was mainly controlled by three key soil properties: acidity, clay, and organic carbon contents. Thus, altitude-specific land-use management holds significance in the distribution of available soil micronutrients in hilly ecosystems.

## Introduction

The soil fertility always enhances crop production and provides sustainability to development opportunities to any Nation. Due to anthropogenic disturbances, the soil has been degraded (in physico-chemical and biological status) and has become one of the global problems. This is more severe in the developing countries of the tropical hilly region having the mountainous gradient^1–3^, where deforestation and conversion of native forests to unsustainable traditional agriculture practices (shifting and settled cultivation along the hill slopes) are rampant. Such land use transformation in the tropical mountainous regions causes loss of top fertile soils carrying loads of nutrients (macro and micro) by soil erosion^4,5^. This adversely affects the crop production, land productivity, and soil sustainability in such hill ecosystem^6,7^. Soil act as a reservoir for the essential plant nutrients (both macro and micro), without which crops cannot complete their life cycles. Soil macronutrients (primary) mostly nitrogen, phosphorus, and potash are required in large quantities but micronutrients like iron (Fe), manganese (Mn), copper (Cu) and zinc (Zn), though required in small quantities, yet play an essential role in maintaining balanced crop growth and ensures the quality of the produce^8^. Low micronutrient availability in soils may lead to sub-optimal plant productivity as well as poor quality of the produce. Plant availability of soil micronutrients is influenced by many factors such as land use change, soil management, and cropping system practices^9,10^. Soil properties such as pH, clay content, organic matter, and other nutrients also significantly influence soil micronutrient availability^8,11^. The solubility of micronutrients in soils is again regulated by several competing reactions such as sorption, precipitation, and even chelate formation. Thus, from an agricultural point of view, ‘the plant available’ form is more important than the total content of micronutrients in soil^12^.

Northeast India (NEI) is a unique ecosystem in the world, with an undulating landscape that extends from < 10 m to beyond 7800 m elevation from mean sea level (masl). Setting in the high rainfall, fragile hilly (> 77% GA: geographical area of 26.2 M ha) ecosystem of the eastern Himalaya (EH), the region is known for its richness in biodiversity, forest covers (in 62% GA), and phytomass^13,14^. Despite significant forest cover, NEI is also highly vulnerable to land degradation attributed to faulty land use practices, strong soil acidity (> 53% GA), and water erosion (> 23% GA). Over the past few decades, deforestation in the form of burning of vegetation due to the prevalence of shifting cultivation (SC), cultivation along the steep slopes in uplands (bun and terrace agriculture), and extensive open cast coal mining converted significant forest areas to degraded scrubland (> 11% GA). This resulted in colossal loss of phytomass and vegetative cover while increased the severity of erosion and soil losses many folds (10 t ha^−1^ to 155 t ha^−1^) in undulating uplands^13,15^. Soils of the region mostly developed from acidic parent materials of shale and sandstone origin in hills while alluvium deposits in the intermountain valleys. Over the years, attention was given to acid soil amelioration through liming, and inorganic fertilization with special emphasis to address P-deficiency while management of micronutrients was almost ignored. As a result, micronutrient deficiency has equally become a major factor of productivity constraints to most of the crops grown in the region. Presently, > 35% soils of the region are deficient in DTPA-extractable Zn, and so as Cu in 1.88% followed by Mn in 1.66% and Fe in 1% area^8^.

Land use land cover change (LULC) often causes alterations of soil properties^1–3,5^ and so, thus, accumulation and plant availability in micronutrients^16^. Such large-scale transformation of forests to agriculture (shifting and settled) and plantation, forests, and agriculture to scrubland in NEI is expected to influence micronutrient availability as well. Previous studies reported land use and altitudinal variation mediated significant changes in soil organic carbon (SOC: 0.5% to 5.5%) in the region^4,13^. Since SOC pool is the primary source of plant nutrition to support marginal input-intensive rainfed agricultural production system of the region, therefore, the concentration of plant-available micronutrients is also expected to vary with land use and altitude mediated changes in soil properties, climate (rainfall, temperature), and vegetation^1,4,7,17^. However, the region still lacks detailed information on complex interaction of acidity, soil properties and interaction of LULC and altitudinal gradient on micronutrient’s availability in this vast (> 26 M ha area) hill ecosystem of Eastern Himalaya. This has become a major bottleneck for devising location-specific micronutrient management strategies for improvement of crop productivity and quality of the produces while restoring degraded scrublands.

Therefore, in the present study, an attempt was made to answer the following key questions:

(i) What is the status of plant-available micronutrient (DTPA extractable Fe, Mn, Cu, and Zn) concentrations in the soils of Northeast India;
(ii) What is the impact of land use change on plant-available micronutrient concentrations;
(iii) How strongly does the altitudinal variation influence the micronutrient concentrations among and across the land uses.

## Materials and methods

### Study area- location, climate and soil

**Figure 1:**
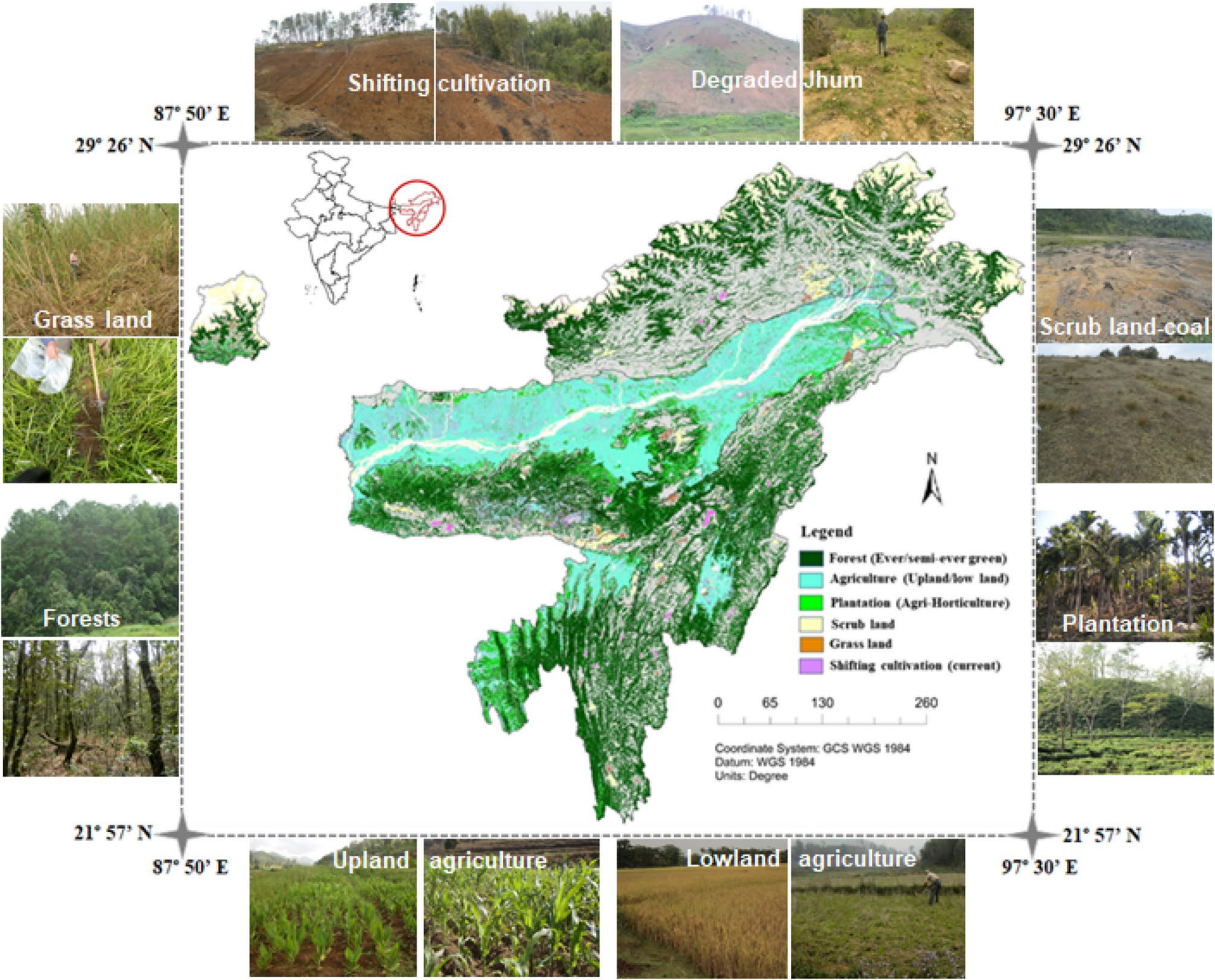
Landuse map of the study area (North-eastern Region of India) derived from multi-date Resourcesat‐2 ortho-rectified LISS‐III satellite data of 2015‐16 in Arc-GIS 10.2. Adapted from National Remote Sensing Centre, ISRO, 2019, all rights reserved. (https://bhuvan-app1.nrsc.gov.in/thematic/index.php).

The study area represents the North-eastern Himalayan region of India (NEHR), lies from 21.57° N to 29.26° N latitude and 87.50° E to 97.30° E longitude with a geographical area (GA) of 26.2 million ha in the fragile Eastern Himalayan (EH) landscape (Fig. 1). Nearly 77% of the region is hilly with steep slopes along an altitudinal gradient extending from < 10 masl (at South Garo Hills, Meghalaya) to above 7800 masl (at Sikkim Himalaya). The varied physiographical features and altitudinal differences in the region give rise to varied types of climate ranging from hot humid to temperate and alpine. Annual average rainfall exceeds 2000 mm with wide orography led spatial variability (1500–11,500 mm)^18^. The region experiences hot summer and cold winter with temperatures varying from sub-zero (during winter) in Sikkim Himalaya to 38° C in the plains (Tripura)^4^.

Soils of the region were mostly developed from the transported materials of shale, sandstone, and recent alluvium deposits^19,20^. In the central plain including Brahmaputra valley (Assam), alluvium derived deep medium-textured soils (loamy) dominate while in the vast hilly region and sloppy uplands (> 77% GA), soils developed from shale and sandstone are red and lateritic with very shallow (in steep slopes) to medium in depth and relatively fine in texture^2,19^. Soils are invariably acidic in reaction, with half of them (53% of GA) are very strong to strong in reaction (pH: 4.5–5.5). Complex interaction of geographic location, high rainfall, and conducive temperature favours luxurious phytomass production which in turn adds higher organic carbon (98% GA with > 1% SOC) in the soils of the region^13^.

### Sampling strategy: selection of sampling locations across land use systems

The LULC map at 1: 50,000 scales of the study area (Fig. 1) was sourced from the Bhuvan web mapping service of the National Remote Sensing Centre, originally derived from multi-season Resourcesat‐2 ortho-rectified LISS‐III satellite data of 2015‐16^14^ and modified (mosaicing individual map of the eight NEHR state into one map representing 8 NEHR states together, Fig. 1) in Arc-GIS v10.2. Nearly 62.5% GA is under different types of forest covers (e.g. Evergreen / semi-evergreen / deciduous /forest plantation) and a half (30.7% GA) of them is evergreen in nature (EF). EF is native forests dominated by subtropical pine (eg. *Pinus spp.*), broad-leaved forest (*Castanopsis indica* Roxb., *Quercus serrata* Murray., *Michelia oblonga* Wall etc.) and Tropical wet semi-evergreen forest (eg. *Shorea robusta* Gaertn. f., *Mesua ferrea* Linn). Deforestation (from jhum, bun agriculture, and coal mining) transformed significant forest areas (> 11% GA) into scrubland (SL). The area under settled agriculture (SA) in upland, lowland paddy (LP), and current fallow occupies 16.2% GA. The agricultural crops in SA are mostly dry-seeded rice, maize, turmeric, and ginger in 2.81% GA while in LP, the puddled transplanted rice-fallow system is practiced in 13.38% GA. Shifting cultivation (SC, jhum) is practiced in 1.20% GA (excluding abandon jhum area) along the steep slopes of uplands, which were previously forests and currently under the cultivation of multiple agricultural crops (rice, maize, tapioca, yam, turmeric, and ginger). Grassland (GL)in 3.3% GA (Table 1) spread across alpine to tropical regions and are dominated by wild grass species like *Setaria sphacelata* Moss., *Panicum maximum* Jacq., and *Thysanolaena maxima* Roxb., with scattered trees/shrubs like *Eupatorium odoratum* Linn., *Ageratum conyzoides* L.^4^. Plantation and horticulture (PH) occupies 2.04% GA with the dominance of tea, rubber, coconut, areca nut, pineapple, citrus fruits, and other unmanaged fruit orchards.

**Table 1:** Information on percent area, sampling numbers, agro-physical variables and measured soil properties (Mean ± SE) under major land use systems sampled across the study area (NER of India).

LULC*	SC	SA	LP	EF	GL	SL	PH
% of TGA^&^	1.20	2.81	13.38	30.68	3.28	11.18	2.03
No. of samples	83	90	98	96	70	93	70
Temperature/ °C	20.7 ± 0.23 (16.5–24.7) ^$^	21.5 ± 0.18 (19.5–25.3)	22.9 ± 0.20 (16.5–27.4)	18.2 ± 0.54 (7.0–25.74)	16.6 ± 1.21 (0.8–25.2)	21.0 ± 0.25 (10.5–25.7)	22.4 ± 0.38 (10.5–26.7)
Rainfall/ mm	2916 ± 17.3 (1200–3850)	2459 ± 5.9 (2100–3500)	2448 ± 63.8 (1280–3500)	2947 ± 25.9 (1234–3603)	2815 ± 57.9 (2200–4800)	2998 ± 150.3 (1635–11,276)	2733 ± 172.7 (1280–11,351)
Altitude/ m	1140 ± 75.6 (931–1809)	1017 ± 6.8 (230–1786)	870 ± 63.3 (20–2000)	1274 ± 21.4 (440–2024)	1742 ± 116.3 (788–4100)	1358 ± 23.1 (1000–1748)	850 ± 90.6 (14–1800)
Soil Order	Ultisol > Inceptisol	Inceptisol > Ultisol	Ultisol > Alfisol > Inceptisol	Ultisol > Inceptisol > Entisol	Ultisol > Inceptisol > Entisol	Ultisol > Inceptisol	Inceptisol > Ultisol > Entisol

*LULC: Treatment abbreviations are explained in text; ^$^: Figures in parenthesis are range of distribution; ^&^TGA = Total geographical area (TGA: 26.22 million ha)

From the map, we identified seven major land use systems dominant in the region and they are as follows (i) Shifting cultivation (SC: current 0–1 year-old); (ii) Settled agriculture (SA) in upland; (iii) Lowland paddy-fallow agriculture (LP); (iv) Natural forest (EF: evergreen/semi-evergreen); (v) Grassland (GL); (vi) Scrubland (SL: including scrub forests, coal mine degraded forests, abandoned degraded jhum); and (vii) Plantation and horticulture (PH) in sloppy uplands. A total of six hundred (600) sampling locations were identified randomly and from each location, a composite of three surface soils (0–15 cm depth) (approximately 500 g) were collected during the post-monsoon rainless dry season (January to April) across the seven land uses along the altitudinal gradients (14 to 4010 masl) (Table 1). Distribution of samples across land uses with a detailed description of agro-physical variables^4^and soil properties analyzed (LULC, percent GA, rainfall, temperature, altitude, particle size distribution, and soil organic carbon—SOC content) under each land use is presented in Tables 1 and 2.

**Table 2:** Soil properties measured across landuses in the NEI.

LULC*	Sand/ %	Silt/ %	Clay/ %	SOC/ %	pH
SC^&^	56.9a^#^ ± 1.05 (23.8)*	17.8b ± 1.04 (33.8)	25.3b ± 0.84 (24.8)	1.73b ± 0.10 (32.7)	4.49b ± 0.08 (13.3)
SA	59.9a ± 0.40 (22.8)	13.9c ± 0.34 (28.9)	26.2 b ± 0.43 (19.1)	1.59b ± 0.04 (27.2)	4.43b ± 0.04 (12.2)
LP	43.4c ± 1.06 (23.6)	21.2a ± 0.46 (20.9)	35.4ab ± 0.99 (27.2)	2.39a ± 0.11 (32.8)	4.94a ± 0.09 (9.3)
EF	39.9 cd ± 2.08 (24.7)	24.7a ± 1.37 (30.0)	35.4ab ± 1.53 (31.2)	2.10a ± 0.12 (33.8)	4.60b ± 0.04 (14.1)
GL	42.5c ± 1.98 (24.9)	18.2b ± 1.42 (24.7)	39.2a ± 2.86 (51.5)	2.26a ± 0.15 (34.3)	4.93a ± 0.09 (6.0)
SL	60.8a ± 1.17 (37.6)	17.5b ± 0.67 (35.4)	21.7b ± 0.73 (30.9)	1.30c ± 0.08 (34.6)	4.10c ± 0.07 (20.0)
PH	51.6b ± 1.84 (27.2)	22.1a ± 0.46 (20.8)	26.2 b ± 1.08 (31.2)	1.22c ± 0.08 (29.7)	4.20c ± 0.05 (11.7)

#: Means in the column followed by different letters (a-d) are statistically significant at *p* < 0.05. *Figures in parenthesis are coefficient of variations (CV) in %. SC^&^: Shifting cultivation, SA: Settled agriculture, LP: lowland paddy, EF: Evergreen forest, GL: Grassland, SL: Scrubland, PH: Plantation and horticulture.

### Soil analysis

The collected soil samples were air-dried and ground to pass through 2.0 and 0·5-mm sieves. Samples sieved through a 2.0-mm sieve were used for soil textural analysis using International Pipette Method^21^ while 0·5 mm sieved samples were analyzed for SOC estimation by Walkley and Black method^22^. Soil pH, cation exchange capacity (CEC), and available macronutrients were determined following standard procedures^23^. The micronutrient estimation was done following^24^ method. Twenty milliliters of 0.005 mol L^−1^ DTPA (Diethylene triamine pentaacetic acid) + 0.1 mol L^−1^ TEA (triethanolamine) + 0.01 mol L^−1^ CaCl_2_ (at pH ~ 7.30) was added to 10 g soil. The solutions were shaken for two hours at room temperature, centrifuged, and filtered through Whatman No. 42 filter paper. Clear aliquots were then analyzed for DTPA extractable micronutrient (Fe, Mn, Zn, and Cu) contents using atomic absorption spectrophotometer (Model Perkin Elmer AAnalyst 200) (Tables 2 and 3).

**Table 3:** Measured DTPA-extractable micronutrient contents (without outliers) under major land use systems in the soils of NEI.

Land uses	DTPA-Fe	DTPA-Mn	DTPA-Cu	DTPA-Zn
	(mg kg^−1^)			
Shifting cultivation (SC)	40.17 (± 3.46)bc*	13.35 (± 1.63)c	0.81 (± 0.07)c	1.42 (± 0.11)bc
Upland agriculture (SA)	35.0 (± 1.70)c	6.44 (± 0.26)d	0.68 (± 0.03)cd	1.25 (± 0.09)bc
Lowland paddy (LP)	52.26 (± 3.24)b	23.79 (± 0.72)b	1.03 (± 0.05)bc	1.63 (± 0.05)bc
Evergreen forest (EF)	48.71 (± 4.31)bc	30.46 (± 2.02)a	1.74 (± 0.06)a	2.13 (± 0.13)b
Grassland (GL)	48.33 (± 2.63)bc	24.91 (± 2.14)b	1.16 (± 0.07)b	3.01 (± 0.10)a
Scrub land (SL)	84.27 (± 4.61)a	5.91 (± 0.42)d	0.59 (± 0.04)d	0.68 (± 0.08)d
Plantation and Horticulture (PH)	76.11 (± 4.48)a	17.80 (± 0.54)c	0.68 (± 0.05)cd	1.06 (± 0.043)c
Over all of the region	58.76 (± 1.72)	20.37 (± 0.753)	1.09 (± 0.045)	1.78 (± 0.115)

*Means in the column followed by different letters (a-d) are statistically significant at 5% level of significance.

### Statistical analysis

Prior to factor analysis, data on DTPA-extractable micronutrients (Table 1) were checked for normal distribution. Analysis of variance was performed using PROC GLM procedure of SAS Version 9·2 to determine the statistical significance of land use effects on micronutrient concentrations. Pearson’s correlation coefficient was used to determine the strength of relationship among the land use, soil properties, and altitudinal variations. Duncan’s multiple range test was done to test the significance of differences between means at *p*-value < 0·05. To reveal the likely causes of the evident differences in micronutrient concentrations among the land use, we computed Pearson correlation coefficients between each of DTPA-extractable micronutrients Fe (Y1), Mn (Y2), Cu (Y3), and Zn (Y4) and the agro-physical parameters (including soil properties). Several of the correlations appeared to be strong, and to rank them in order of importance, we did step-wise multiple regressions of Y1, Y2, Y3 and Y4 as response variables on the agro-physical and soil parameters (including macronutrients—NPK) as predictors. We judged the predictors significant if the probability that their contributions were null was < 0.05, i.e. (*p* < 0.05 of the null hypothesis).

## Results and discussion

### Agro-physical variables and soil properties in the study area

Altitudinal variations in the sampling locations ranged from 14 masl (in Garo Hills, Meghalaya) to over 4050 masl (in Sikkim Himalaya) (Table 1). Dense evergreen forest (EF) dominated the region from an elevation beyound 400 m till 2100 m and then grasslands (GL) extended to 4100 m in the alpine snow-clad Sikkim Himalaya. The forest degradation to scrublands (SL) by coal mining and shifting cultivation (SC) mostly occurred at an elevation range of 900 m to 1850 m in the region. Settled agriculture (SA and LP), and plantation crops (PH) were cultivated in wide elevation ranges from 14 to 2000 masl (Table 1). Mean annual temperature varied widely from 0.8° C in high—altitude (> 4000 m, Sikkim) GL to 27.4° C in low altitude LP (< 40 m, Assam). Similarly, annual rainfall also varied from 1200 mm (in Nagaland) to 11,351 mm in Cherrapunji Plateau (Meghalaya) (Table 1). Soil particle size distribution (PSD: sand, silt, and clay) varied widely (coefficient of variation, CV: 19.1% to 54.7%) across the study area (Table 2). The sand was dominant (> 50% to 60.8%) in SL, SA, SC, and PH while silt + clay dominated (56.6–61.1%) PSD in EF, GL, and LP. Among the land uses, EF, GL and LP had significantly (*p* < 0.05) higher clay contents (> 35% to 39.2%) than SL, SA, and SC. Soils were high in mean SOC content (1.22% in PH to 2.26% in GL) but varied widely (CV: 27.2% to 54.6%) across land uses. Average SOC content was significantly (*p* < 0.05) higher (> 2.0%) in GL, EF, and LP than other land uses. Invariably, soils of the studied region were moderate to strongly acidic in reaction (pH: 3.05 to 6.43) with low-intensity variation (CV < 25%) but more acidified under SL and PH (pH: 4.10–4.20) than other land uses (Table 2).

Wide altitudinal gradients (< 9 masl in Meghalaya to above 7800 masl in Sikkim Himalaya) in Indian Eastern^2,4^ and Western Himalayan region results in variable agrophysical properties namely climate (rainfall and temperature), sand, silt, clay, and organic carbon contents in the region^2,3,17,25^. Non-linear increase in rainfall, silt + clay, and organic carbon while a decrease in temperature and sand along an altitudinal gradient (< 500 to 3500 m) was reported by earlier workers as well^4,13,17,25^. Land uses and vegetation composition plays a significant role in contributing to textural variation and SOC contents in the soils of Indian Himalaya^25^. Soils in the region change very frequently within short distances which in turn change the vegetation pattern also^1,3,26^. The dominance of sands and low SOC content (< 1.6%) in upland hill agriculture (SA and SC) and scrublands while higher clay and SOC contents (> 2.0%) in non-agricultural land uses (forests and grasslands) were reported in Indian Himalaya^1,4,6^. Cultivation practices along the hill slopes in UA and SC with the burning of vegetation degrade soil structure, reduce the aggregate stability and make the soils more prone to runoff and other forms of erosion^3,4,25^. The non-agricultural land uses (EF and GL) being located at higher altitude, receiving higher annual rainfall with low mean temperature favoured higher phytomass production and accumulation of higher organic carbon by reducing oxidation rate^3, 17,26^, producing better aggregate stability, and thus reducing the loss of SOC by soil erosion^4^. Higher clay contents might have further helped in the accumulation of SOC^3,7,25^ because clay reflected a significant positive coefficient and correlation with SOC concentrations.

### Variability in micronutrient concentration and land use effect

**Figure 2:**
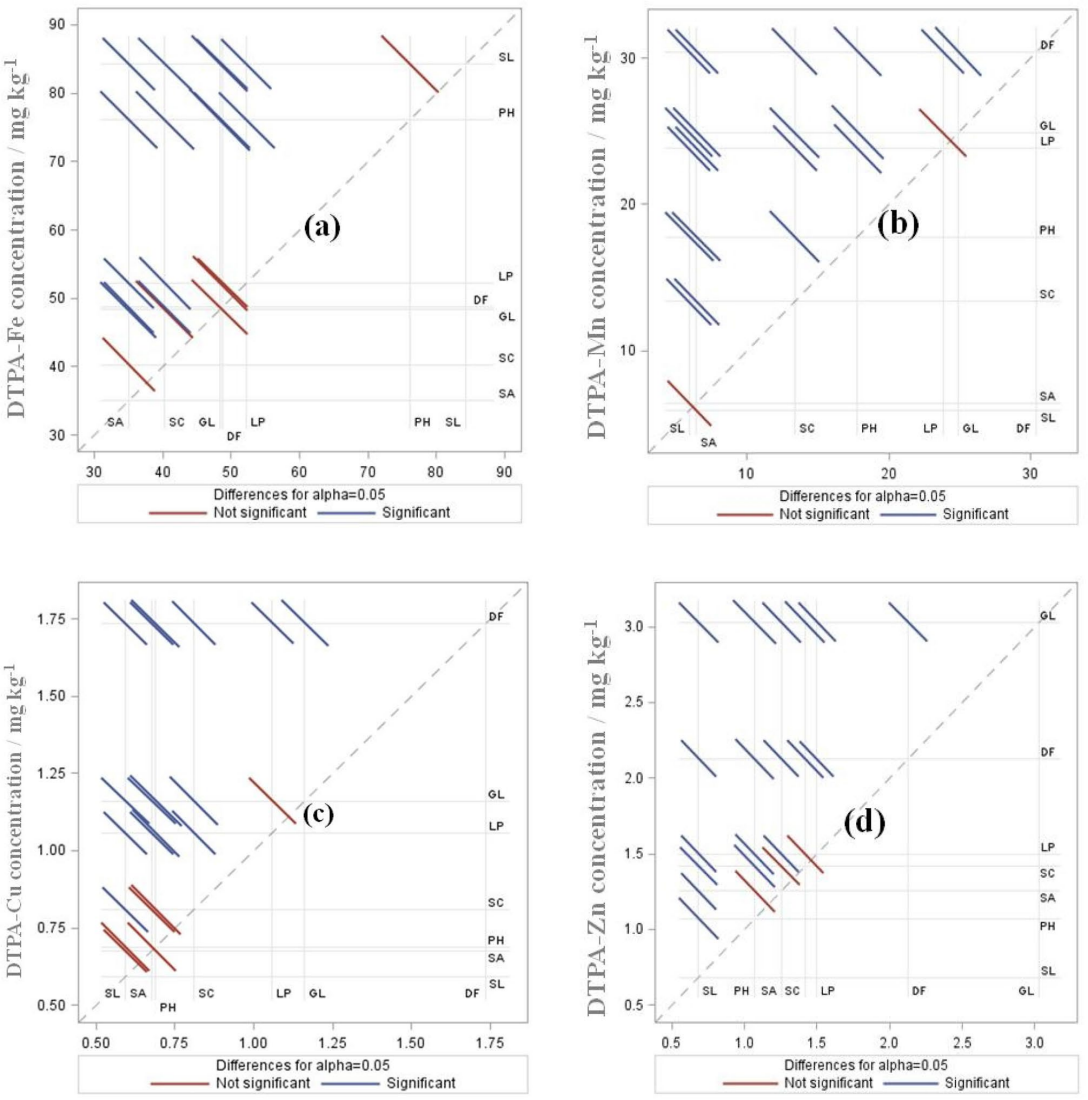
Diffograms showing significant differences among the seven land uses in available Fe, Mn, Cu and Zn contents. Bold horizontal and vertical lines represent the seven land uses of North-east India. The small lines passing through the squares diagonally represent non-significant or significant differences between the two corresponding land uses. Treatment abbreviations are *SC* shifting cultivation, *SA* settled agriculture, *LP* lowland paddy, *EF* evergreen forest, *GL* grassland, *SL* scrubland, and *PH* plantation and horticulture.

The DTPA-extractable Fe concentration across the study area varied widely from 0.665 to 257.1 mg kg^−1^ while Mn, Cu, and Zn concentrations ranged from traces to 93.4, 17.1, and 34.2 mg kg^−1^, respectively. Mean Fe, Mn, Cu, and Zn concentrations were 58.76, 20.37, 1.09, and 1.78 mg kg^−1^, respectively. The effect of landuse changes on micronutrient concentration was significant (*p* < 0.05) in the studied soil (Table 3). Soils under non-cultivated EF and GL had comparable mean Fe concentrations (48.3–48.7 mg kg^−1^). On cultivation in uplands (SC and SA), mean Fe concentration decreased (− 17.7 to − 28.1%) while LP had a comparable concentration with EF. However, on the degradation of EF to SL, Fe concentration increased (+ 73%) and recorded the highest (Fe: 84.3 mg kg^−1^) followed by plantation (PH) in sloppy uplands (+ 56.2% higher than EF). However, EF recorded the highest Mn (30.5 mg kg^−1^) and Cu (1.74 mg kg^−1^) concentrations followed by GL. Conversion of forests to upland agriculture and PH significantly (*p* < 0.05) reduced the Mn (− 41.5 to − 78.8%) and Cu (− 53.4 to − 60.9%) concentrations. On the degradation of EF to SL, concentrations of Mn and Cu further decreased and recorded the lowest among all the seven land uses. The mean Zn concentration was significantly (*p* < 0.05) higher (2.13 to 3.0 mg kg^−1^) in non-cultivated (GL > EF) than cultivated land uses (SC, SA, LP, and PH). The reduction in Zn concentration was more in PH (− 50%) and upland agriculture (− 33 to − 41%) than lowlands (− 23.5%) over EF. Scrubland recorded the lowest mean Zn concentration, 68% less than EF (Table 3; Fig. 2).

The variability in micronutrient concentrations were expected due to the highly variable nature of altitude (from < 15 to > 4000 masl) mediated climate (tropical to alpine) with wide spatial variability in mean annual rainfall (from 1200 to over 11,300 mm), temperatures (0.8 to 27.4° C), vegetation types from different land use practices^1,3,26^, and heterogeneous soil properties^7^ including acidity (pH: 3.05–6.43), clay (< 10 to > 72%), and SOC contents (< 0.5 to > 5.0%)^4,13^. Previous studies from the region (coal mine degraded Jaintia Hills, Meghalaya)^27^ reported a variation in average DTPA-Fe concentration from 84 to 260 mg kg^−1^ while average DTPA-Mn varied from 7.2 to 13.5 mg kg^−1^. Similarly, Shukla and Behera^28^ reported a wide variation in DTPA- Mn, Cu and Zn concentrations from traces up to 445, 136 and 52.9 mg kg^−1^, respectively in different soil types of India. Our reported values of micronutrients (Table 3) fall within the reported ranges of Shukla and Behera^28^. Available micronutrients in the strongly acidic soils (pH < 5.5) of hilly ecosystem of NEHR developed from acid igneous rocks under alpine to tropical climate was in the order of Fe > Mn > Zn > Cu. Our trends of Fe, and Mn dominancy was mostly attributed to strong acidity (pH < 5.0) induced release of hydrous oxides of aluminum, Fe and Mn from the igneous parent materials (mostly sandstone origin) under tropical climate of the region^29^.

With each unit increase in soil pH from 4 to 9, the solubility of Fe in soil decreases by 1000-fold while it decreases 100-fold for Mn, Cu and Zn^30^. We also observed a strong negative correlation of all four micronutrients with soil pH (r =  − 0.05 to − 0.255). The soils from areas with higher clay in particular and SOC to some extent also had significantly higher micronutrient concentrations. Correlation studies also affirmed a strong positive relation of micronutrients with clay (r =  + 0.171 to + 0.507) and SOC (r =  + 0.103 to + 0.230) contents. Step-wise multiple regression analysis also revealed that besides rainfall and elevation, three soil variables, namely clay, SOC, and pH significantly (*p* < 0.05) influenced the availability of micronutrients (Table 4). Parameter estimation reflected steep and positive slopes of clay while negative of pH with all four micronutrients, suggesting that with an increase in clay content and decrease in pH, micronutrients increased significantly (Table 5). Soil pH strongly influences Zn availability and in acid soils, the influence is greater^28^. Similarly, SOC reflected positive slopes with Cu and Zn, suggesting that increase in SOC content significantly influenced Cu and Zn concentrations (Table 5).

**Table 4:** Pearson’s correlation matrix between soil properties (particle size, pH, and SOC) and DTPA micronutrients (Fe, Mn, Cu and Zn/ mg kg^−1^) in the soils (0–15 cm) depth across NE India.

Parameter	Sand	Silt	Clay	pH	SOC	Fe	Mn	Cu	Zn
Sand/%	1.000								
Silt/%	− 0.561**	1.000							
Clay/%	− 0.847**	0.108**	1.000						
pH	− 0.127**	0.125**	− 0.007	1.000					
SOC/%	− 0.221**	0.156**	0.234**	− 0.123**	1.000				
DTPA-Fe	− 0.167**	0.115**	0.171**	− 0.255**	0.119**	1.000			
DTPA-Mn	− 0.267**	0.206**	0.204**	− 0.171**	0.230**	0.106*	1.000		
DTPA-Cu	− 0.244**	0.115**	0.242**	− 0.049	0.103**	0.061	0.424**	1.000	
DTPA-Zn	− 0.358**	− 0.116**	0.506**	0.111**	0.222**	0.020	0.439**	0.455**	1.000

*Significant at *p* < 0·05 level of significance (r = 0.0735); **Significant at *p* < 0·01 level of significance (r = 0.103).

**Table 5:** Parameters of multiple regression of DTPA extractable micronutrient contents—Fe (Y1), Mn (Y2), Cu (Y3) and Zn (Y4) of elevation and soil variate of NE soils: clay, pH, SOC (Y1 = a0 + a1 × elevation + a2 × clay + a3 × pH + a4 x SOC); (Y2 = a0 + a2 × clay + a3 × pH + a4 × SOC); (Y3 and Y4 = a0 + a1 × elevation + a2 × clay + a3 × pH + a4 × SOC). The effect of climate variables (rainfall, temperature), other soil properties (bases, nitrogen, phosphorus, potash, sulfur, soil BD etc.) were marginal (non-significant at *P* < 0.05), so they were not included in the model. The residual degrees of freedom for Fe, Mn, Cu, and Zn are 551, 551, 557, and 555, respectively and the explanatory variables were significant at *P* < 0.01.

Parameter	DTPA-Fe	DTPA-Mn	DTPA-Cu	DTPA-Zn
	mg kg^−1^			
F-ratio	19.8_(df−4,551)_	22.3_(df−3,551)_	11.9_(df−4,557)_	25.1_(df−3,555)_
a_0_	− 46.701	4.752	1.125	0.339
a_1_	0.02	ns	0.03	0.0003
a_2_	0.367	0.265	0.007	0.0091
a_3_	− 6.329	− 0.122	− 0.011	− 0.023
a_4_	0.07	0.12	0.067	0.005
R^2^_adj_	59.9	53.9	55.9	63.6

ns: non-significant at *p* ≤ 0.05.

The constructed diffograms among the seven land uses (Fig. 2) affirmed significant (*p* < 0.05) effect of land uses on micronutrients. Scrublands were earlier under forests in upland steep slopes. However, due to continuous deforestation in the form of open cast coal mining, jhumming, and bun-agricultures (along the steep slope) and on repeated re-cultivation on post-jhum short fallow periods (2–3 years) transformed them into degraded land^4,14,27^. The abandoned jhum lands (after 2–3 years of SC) in steep slopes losses top fertile soil layers from runoff and soil erosion in this high-intensity rainfall zone (> 3500 mm annually). Another reason is deforestation from large-scale open cast coal mining in the scrub forests followed by seepage and influx of acid mine drainage (AMD). The sulfur (12%), mineral contaminants (biogenic pyrites, marcasite, and trace metals), and heavy metal-rich AMDs (mostly iron-rich pyrites, aluminum oxides) on oxidation acidified the soils further (pH: 3.05–4.0) and contributed large concentrations of Fe^27,31^. Despite strong acidic soil reaction, lack of Mn, Cu and Zn-containing minerals (sphalerite) in the coal deposits, partially from abundances of pyrites and aluminum oxides^27^ and loss of top fertile soils (occasionally exceeds 100 t ha^−1^ yr^−1^) in degraded jhum lands^15^ might have resulted in poor Mn, Cu, Zn concentrations^32^ than forests and grasslands. We also recorded > twofold higher soluble aluminum (AL) (> 36 mg kg^−1^) in SL over EF and GL (14–17 mg kg^−1^). Despite burning of vegetations, soils under current jhum (SC) had significantly (*p* < 0.05) higher micronutrients (Fe, Mn, and Zn) than the soils from sloppy upland agriculture (SA). Generally, deterioration led by soil erosion and loss of top fertile soils in jhum starts from second to third years of continuous cultivation of multiple crops and continues beyond 2–3 years of post-jhum fallow periods. On re-cultivation after short fallow cycles (2–3 years), runoff (20–39% of total rainfall) and soil loss becomes more severe (19.16–155.5 t/ha annually) in the region^33,34^. In our SC areas, soils with freshly burned forests/vegetative covers were exposed to only one season of rainfall and the soil erosion process might yet to reach the peak to cause soil losses, unlike in degraded abandoned jhum lands in the region under SL^35^. In sloppy SA, cultivation of agricultural crops (mostly nutrient exhaustive maize and turmeric) reduced the concentration of available micronutrients. Repeated soil disturbances (removal of weed biomass and tillage activities) and continuous nutrient mining with complete absence of micronutrient replenishments from external sources^2,4,6^ might have contributed to the decline in micronutrients in the soils under SA. The soils under lowland paddy (LP) although subjected to similar nutrient mining as that of SA, yet, could retain higher organic matter (SOC: 2.39%). Prevailing anaerobic conditions in wetland rice cultivation in LP unlike the dry-aerobic condition in sloppy SA further helped in increasing mobility of micronutrients, particularly while building up organic matter accumulation from a lower rate of oxidation process^36^. Paddy soils also had significantly (*p* < 0.05) higher clay contents (weighted average, > 35%). All these might have contributed to significantly higher micronutrients in LP than upland agriculture (SA and SC).

Non-cultivated land uses (GL > EF) registered higher soil Zn concentration than cultivated (SA, SC, LP, PH) and degraded (SL) land uses. This agrees with the study conducted by Mengiste et al.^37^ in the Gambella Region of Ethiopia, where DTPA extractable Zn was higher in high altitude grazing lands compared to cultivated lands. Grassland soils were rich in clay contents (> 39%), which might have contributed to the higher build-up of Zn since clay content had a strong positive correlation with Zn contents (r =  + 0.506**) in the soil (Table 4). Higher values of zinc (20–34 mg kg^−1^) in GL were mostly measured in yak grazing high altitude (> 3000–4100 masl) alpine region of Sikkim Himalaya. Deposition of yak excreta rich in Zn^38^ at sub-zero, to less than 1° C temperature along with the extensive fibrous root systems of the grasses might have deposited a substantial amount of organic carbon (> 2.20%). Like clay, SOC contents also reflected significant positive correlation (r =  + 0.222**) (Table 4) with Zn contents. Forest soils had higher clay and SOC contents, next to GL while relatively more acidic (0.39 unit less) than GL. Deposition of litter-falls without soil disturbances might have favoured the accumulation of comparable DTPA-Fe, with GL but higher in Mn, Cu, and second-highest Zn contents only next to GL. The scrubland was distinctly apart from other land uses while PH was in the transition zone between EF to SL, relatively closer to SL. Comparable soil properties (clay, SOC, pH, soluble aluminum) including micronutrients in PH and SL might be other reasons. We also recorded a higher concentration of soluble aluminum (32 mg kg^−1^) in PH, comparable to SL while almost double to EF and GL (14–17 mg kg^−1^). The transformation of PH from previously forests (canopy density > 60%) to the cultivation of agricultural plantation (tea, rubber) including poorly managed orchards (citrus peach, pineapple, etc.) in sloppy uplands reduced the canopy density substantially (< 40%) and made them susceptible to soil erosion^3,17,25^.

### Effect of altitudinal gradients on micronutrient concentrations

**Figure 3:**
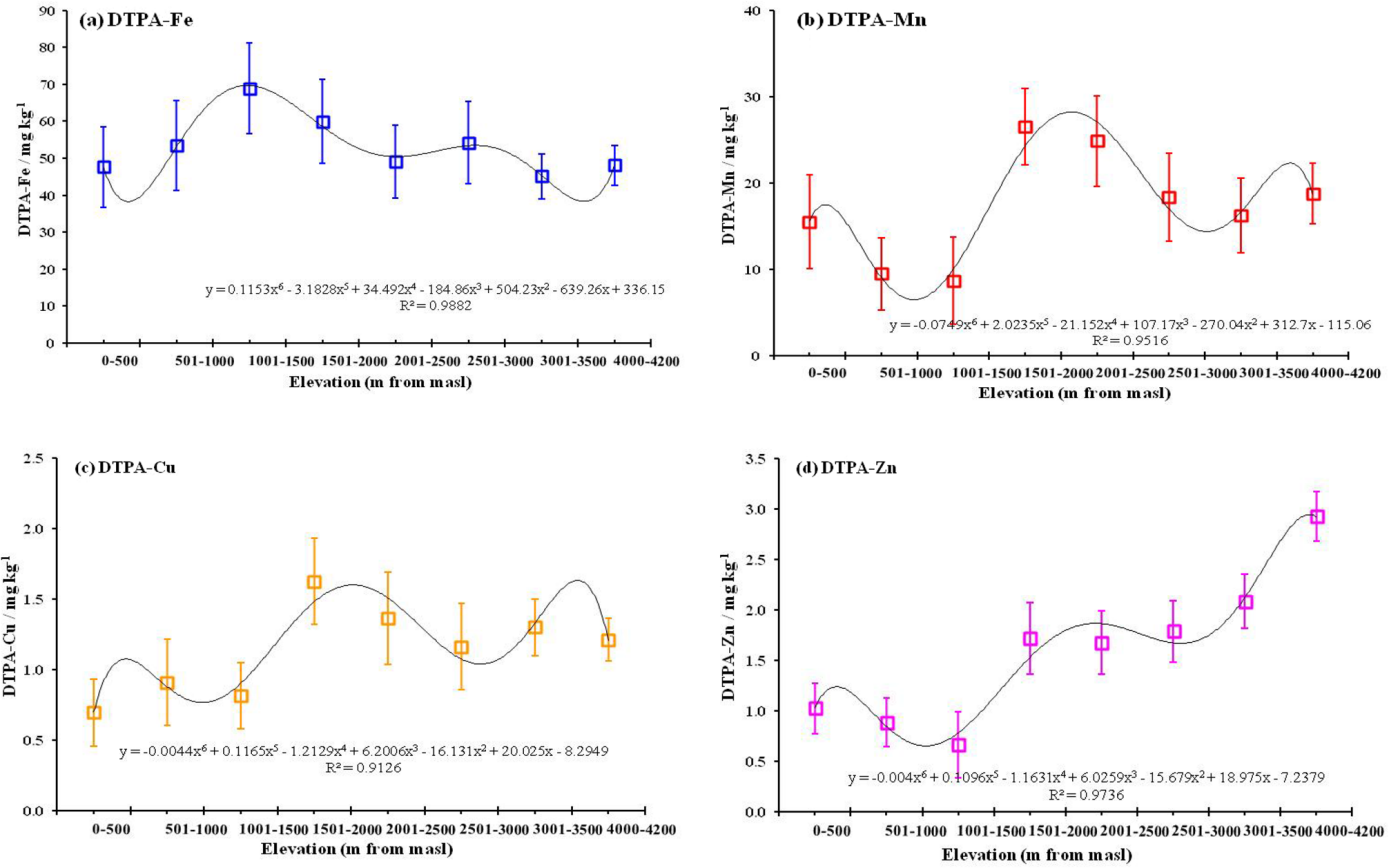
Altitudinal gradients (< 15 m to > 4000 m masl) on DTPA-extractable (**a**) Fe, (**b**) Mn, (**c**) Cu, and (**d**) Zn concentrations. Dotted lines are polynomial trends.

Due to the variable responses of micronutrients to altitudinal gradients ( at an increment of 500 m) under each land use practice, the interaction effect of altitude and land uses (across) was non-linear and followed a complex 6th—order (sextic) polynomial function fit best (R^2^ > 0.934) (Fig. 3). Across all land uses, Fe concentration (averaged of 500 m) increased consistently from the baseline elevation (0–500 m, 47.6 mg kg^−1^) and reached the peak at 1000–1500 m masl (68.8 mg kg^−1^) and then decreased inconsistently with several smaller peaks and valleys (Fig. 3a). Contrary to Fe, Mn concentration decreased consistently from baseline (15.6 mg kg^−1^) till 1001–1500 masl and then increased to reach the peak (26.5 mg kg^−1^) at 1501–2000 masl. Beyound 2000 m elevation, it decreased consistently and increased marginally again at 4100 masl (Fig. 3b). The Cu concentration increased inconsistently from the baseline (0.695 mg kg^−1^) to reach the first peak at 1501–2000 m (1.63 mg kg^−1^) and then decreased inconsistently with smaller peaks and valleys till 4000–4100 masl (Fig. 3c). The Zn concentration, however, decreased consistently from the baseline (1.02 mg kg^−1^) till it reached the 1st peak at 1501–2000 m (1.751 mg kg^−1^) and then increased inconsistently till it reached the highest peak (2.92 mg kg^−1^) at 4000–4100 masl (Fig. 3d).

**Figure 4:**
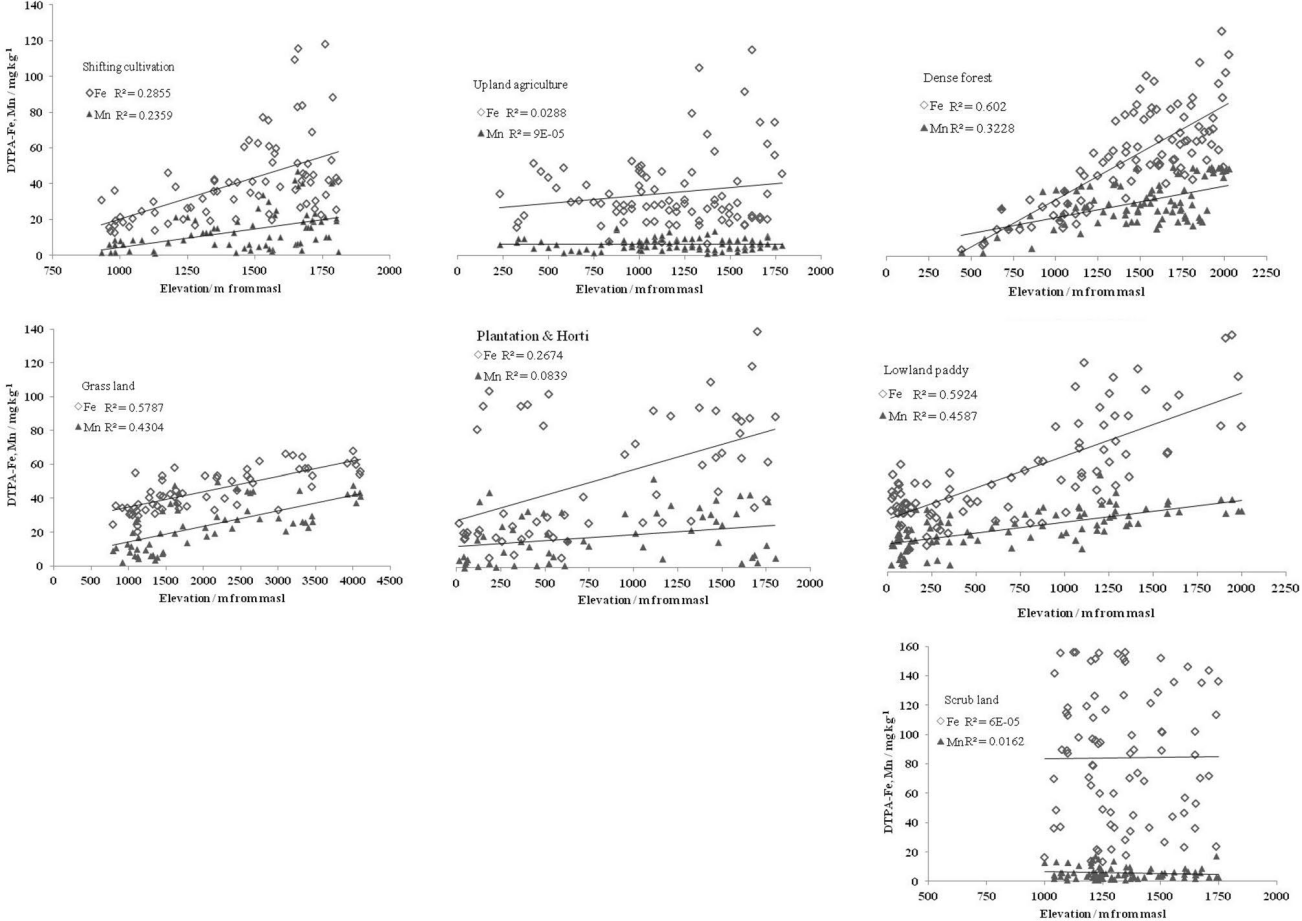
Effect of land uses on response of DTPA extractable Fe and Mn (mg kg^-1^) concentrations along the altitudinal gradients.

**Figure 5:**
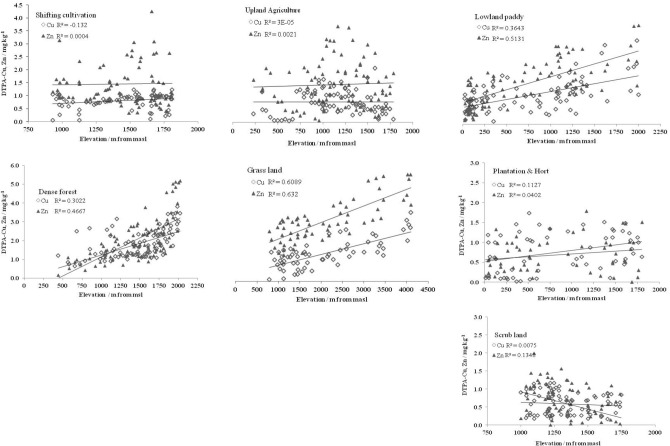
Effect of land uses on response of DTPA extractable Cu and Zn (mg kg^-1^) concentrations along the altitudinal gradients.

Among the land uses, the trends of micronutrients were different along with the altitudinal gradients (Figs. 4, 5). In uplands, only Fe and Mn concentrations increased significantly (*p* < 0.005, r =  + 486 to + 0.534) in SA while non-significant trends of micronutrients with elevation were observed in SA (Figs. 4, 5, Table 6). In contrast, plantations (PH) in upland recorded significant (*p* < 0.05–0.0005, r =  + 0.290 to + 0.517) increasing trend in Fe, Mn, and Cu concentrations with elevation. In SL, the distribution of Fe was scattered and without any trend (Fig. 4) while Mn, Cu, and Zn concentrations decreased with the increase in elevation (Fig. 5, Table 6). However, in non-cultivated EF and GL, all four micronutrients had significant (*p* < 0.0005, r =  + 0.557 to + 0.795) increasing trend with elevations. Cultivated lowlands (LP) was an exception where alike EF and GL, all the four micronutrients increased significantly (*p* < 0.0005, r =  + 0.604 to + 0.770) with elevations (Figs. 4, 5; Tables 1 and 6).

**Table 6:** Response of land use systems to altitudinal gradients measured in terms of correlation coefficients (r) on particle size distribution (%), SOC (%) and DTPA-extractable micronutrient contents (mg kg^−1^).

Altitudevs	Correlation coefficient values (r) with altitudinal gradients						
	SC^&^	SA	DF	GL	SL	PH	LP
Sand /%	0.030	− 0.190	− 0.138	− 0.461	0.248*	0.103	− 0.413
Silt /%	− 0.017	0.018	0.156	− 0.241*	− 0.082	− 0.001	0.096
Clay /%	0.016	0.163	0.327***	0.575****	− 0.001	− 0.218	0.396****
SOC (%)	0.425****	0.214*	0.544****	0.531****	− 0.008	0.303*	0.437****
DTPA- Fe	0.534****	0.170	0.776****	0.761****	0.007	0.517****	0.770****
DTPA- Mn	0.486****	0.009	0.568****	0.656****	− 0.127	0.290*	0.677****
DTPA- Cu	0.166	− 0.005	0.557****	0.746****	− 0.086	0.216	0.604****
DTPA- Zn	0.020	0.046	0.683****	0.795****	− 0.367**	0.201	0.716****

Level of significance at *P* = 0.05 (*), 0.01 (**), 0.005 (***) and 0.0005 (****); SC^&^: Shifting cultivation, SA: Settled agriculture, LP: lowland paddy, EF: Evergreen forest, GL: Grassland, SL: Scrubland, PH: Plantation and horticulture.

In the region, maximum degradation of primary forests to scrubland was experienced in the altitudinal range of 950 to 1500 masl. This was attributed to the presence of large scale open-cast coal mining, short fallow (of 2–3 years) abandoned jhum land, and cultivation in vegetation burned raised beds (buns) across steep slopes (bun agriculture). Soil erosion led to topsoil loss from such land use practices exceed critical threshold limit (12.5 t^−1^ ha^−1^ yr^−1^) by several fold at this altitudinal range. In addition, AMDs from coal mining in selective areas (Meghalaya) contaminated the soils with an excessive amount of oxides and hydroxides of Fe, sulfur (S), and aluminum (Al) several-fold higher than toxic limits. We measured DTPA-Fe concentration 2.5 times higher than toxic thresholds (100 mg kg^−1^), 3.5 folds higher soluble Al (toxic thresholds of 10 mg kg^−1^) and tenfold higher available form of S (toxic thresholds of 20 mg kg^−1^)^27,29^ with extremely acidic soil reaction (pH < 3.5). This might be the reason for registering the highest Fe concentration but lesser amount of the other three micronutrients (Mn, Cu, and Zn) at 1001–1500 m masl. Weak correlation between DTPA-Fe and other three micronutrients (Table 4) also indicated the dominance of Fe marginalized the presence of other micronutrients, mostly due to anthropogenic land disturbances. Other reasons for low concentrations of Mn, Cu, and Zn in the altitudinal range of > 900–1500 m masl is due to the transformation of forests to settled agriculture in high rainfall (> 3000 mm annual) sloppy uplands. Cultivation of erosion permitting crops (maize, zinger, turmeric, root crops, etc.) across steep slopes after clearing of natural vegetations in SA made them vulnerable to erosion led topsoil loss^2,4^. Lack of periodic nutrient replenishments from fertilization/manuring in the marginal input-intensive upland agriculture in the region did not compensate for the losses (via erosion and runoff, nutrient leaching, continuous crop nutrient mining) in soil reserves. This may be attributed to non-significant trends of micronutrients with elevation in SA, comparable to SL (Figs. 4 and 5).

Significant increase in SOC contents (*p* < 0.05–0.005) and more soil acidification (pH decreased by 0.25–0.33 units) at higher altitudes (> 930 m masl) under freshly jhum and plantations with limited soil disturbances might have contributed to the significant (*p* < 0.05–0.005) increasing trend of Fe and Mn concentrations with elevation. However, the dominance of non-disturbed primary forests (EF) and grassland (GL) beyond 1500 m masl resulted in higher concentrations of Mn, Cu (at 1501–2000 m masl), and Zn (GL at 4100–4200 m masl) (Fig. 3b–d). An increase in SOC and clay contents in EF and GL might have caused a significant (*p* < 0.005) increase in micronutrients with elevation. The cultivated lowland (LP) had comparable significant ((*p* < 0.005) trends with EF, mostly due to the increase in clay and SOC contents with elevation (Figs. 4, 5, Table 6). The presence of LP at 1501–2000 m also contributed to a higher Mn and Cu concentrations at 1501–2000 m masl. Presence of GL at high-altitude (> 3000 m masl) alpine region of Sikkim Himalaya resulted in higher concentration of Zn.

In cultivated sloppy uplands and scrublands, a range of unsustainable agricultural practices at different intensities of disturbances (deforestation, burning of vegetations, soil pulverization, and growing of nutrient exhaustive multiple crops, mining) offset the positive influence of altitude mediated changes on soil properties^1,3,17^ including micronutrient concentrations. Altitudinal variation modifies temperature, in turn, controls the decomposition of organic matter and accumulation of SOC^4^. We observed consistent increase in SOC and clay contents only in non-disturbed land uses (EF, GL) along with LP as an exception compared to inconsistent trends in disturbing land uses. As a result, the measured SOC and clay contents in our study followed a non-linear polynomial 5th order function across altitudinal gradient (< 500 m till 4200 m masl). Differences in SOC content and PSD particularly clay contents associated with changes in altitude^2,7^ strongly influence micronutrient distributions in soil^8^.

## Conclusion

We conclude that the plant available micronutrients extracted by DTPA in the acid soils of the Eastern Himalaya (Northeast India) exceeded (6.9 times for Fe, 2.9 times for Mn, 1.36 times for Cu, and 1.48 times for Zn) by far critical limits for Indian soils (8.5 mg kg^−1^for Fe, 7.0 mg kg^−1^ for Mn, 0.8 mg kg^−1^ for Cu, and 1.2 mg kg^−1 ^for Zn). Conversion of forests to upland agriculture (shifting and settled) significantly (*p* < 0.05) reduced concentrations of Fe (− 17.7 to − 28.1%), Mn (− 41.5 to − 78.8%), Cu (− 53.4 to − 60.9), and Zn (− 33 to − 41%) while cultivation in lowland (paddy), the reduction in Mn (− 21.8%), Cu (− 40.8%), and Zn (− 23.5%) was relatively less. The transformation of forests to plantation crops in sloppy uplands also substantially reduced Mn, Cu, and Zn (by 41.5 to 60.9%). Degradation of forests to scrubland from large-scale open-cast coal mining led to acidification followed by metal (Fe) contamination (73% increase) while reduced (by 66.1 to 80.6%) other micronutrients. However, non-cultivated grasslands at high altitudes had comparable Fe, Mn, and Cu but higher (+ 29%) in Zn concentrations than forest soils. Micronutrients responded differently and followed a non-linear, 6th—order polynomial trend along the altitudinal gradients (< 500 m to 4100 m masl). This was mostly attributed to the dominance of specific types of land use (s) at particular altitudinal ranges. Overall, wide variability in plant available micronutrients could well be explained as an outcome of the interaction from land uses and altitudinal gradient mediated changes in key soil properties. The soil properties were acidity (pH), clay, and organic carbon contents, which together contributed 54–64% variation in the soils of the region.
